# Complete pathological response with pembrolizumab in recurrent sigmoid adenocarcinoma

**DOI:** 10.1093/jscr/rjag168

**Published:** 2026-03-21

**Authors:** Benson Li, Truong Ma

**Affiliations:** Department of Surgery, Summa Health System, 141 N Forge St, Akron, OH 44304, United States; Department of Surgery, Summa Health System, 141 N Forge St, Akron, OH 44304, United States

**Keywords:** colorectal adenocarcinoma, immunotherapy, complete pathological response, pembrolizumab, programmed cell death protein-1 inhibitor

## Abstract

Genetic evaluation for mismatch repair deficiency (dMMR) or microsatellite instability (MSI) is now routinely performed as part of the workup of colorectal cancer. The traditional approach to advanced disease was chemotherapy-based agents; however, dMMR/MSI patients were found to respond more poorly. Immunotherapy, such as pembrolizumab, a programmed cell death protein-1 inhibitor, has evolved as a superior option. This report describes a 48-year-old with a history of stage IIC sigmoid adenocarcinoma who developed recurrent uterine and peritoneal implants following surgical resection and adjuvant chemotherapy. Given the concern for recurrent disease, the appropriateness of colostomy reversal remained uncertain. The patient subsequently underwent 20 cycles of pembrolizumab followed by colostomy reversal with concurrent resection of suspected implants. Final pathology revealed a complete pathological response (cPR) with no evidence of residual malignancy. This case highlights the potential for pembrolizumab to induce a cPR in dMMR colorectal cancer despite the absence of radiographic regression.

## Introduction

The treatment of colorectal cancer has become increasingly individualized with the incorporation of advanced genetic testing. Assessment for mismatch repair gene mutations or microsatellite instability is now routinely performed in all colorectal cancer patients, with ~15% demonstrating deficient mismatch repair (dMMR). Traditionally, advanced disease has been managed with systemic neoadjuvant or adjuvant chemotherapy, with or without upfront resection. More recently, immunotherapy has gained significant interest as an alternative to conventional chemotherapeutics, particularly in dMMR patients. Pembrolizumab, a programmed cell death protein-1 (PD-1) inhibitor, has demonstrated superior efficacy and fewer treatment-related adverse events compared with traditional chemotherapy in dMMR colorectal cancer [1]. However, the full therapeutic potential of immunotherapy—particularly its ability to induce a complete pathological response (cPR)—remains under investigation.

We report the case of a 48-year-old woman with a history of stage IIC sigmoid adenocarcinoma who developed recurrent uterine and peritoneal implants following resection and adjuvant chemotherapy. She later completed 20 cycles of pembrolizumab with repeat magnetic resonance imaging (MRI) demonstrating stable disease. However, given the disease recurrence, it was difficult to determine whether colostomy reversal was appropriate in the setting of a possible recurrence. Ultimately, a multidisciplinary conference was held, and she underwent colostomy reversal and resection of the implants, with final pathology demonstrating a complete pathological response.

## Case report

A 48-year-old woman with a history of stage IIC moderately differentiated sigmoid adenocarcinoma (pT4bN0 with local invasion into the distal rectal wall and vaginal sulcus) underwent resection with end-colostomy at an outside hospital 2 years prior. She presented for evaluation for colostomy reversal. Genetic testing at the time of diagnosis revealed loss of MSH6 and a monoallelic MUTYH mutation, consistent with Lynch syndrome in the setting of her family history.

**Figure 1:**
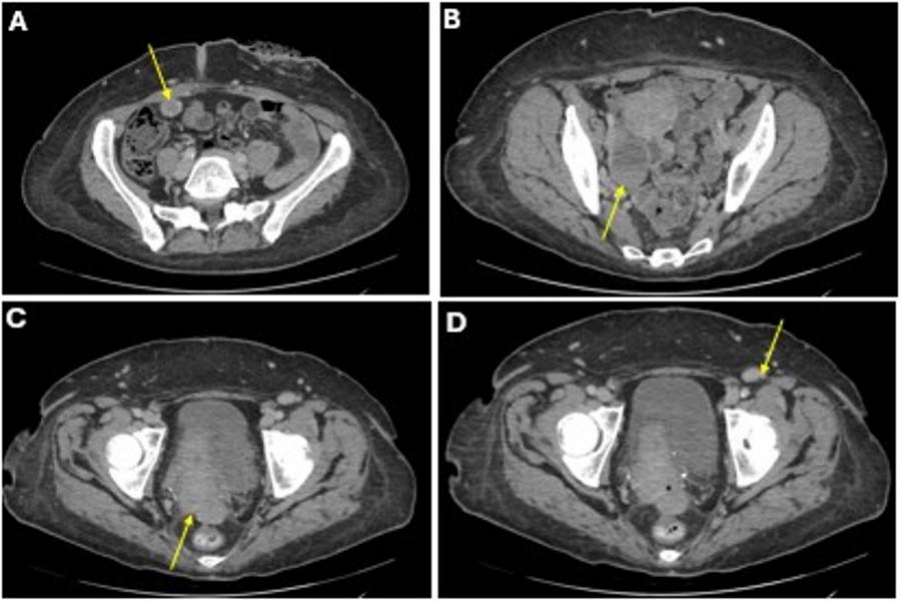
A CT imaging showing lesions involving the right abdominal wall (A), right ovary (B), right anterior reflection of the rectum (C), and left inguinal lymph node (D).

**Figure 2:**
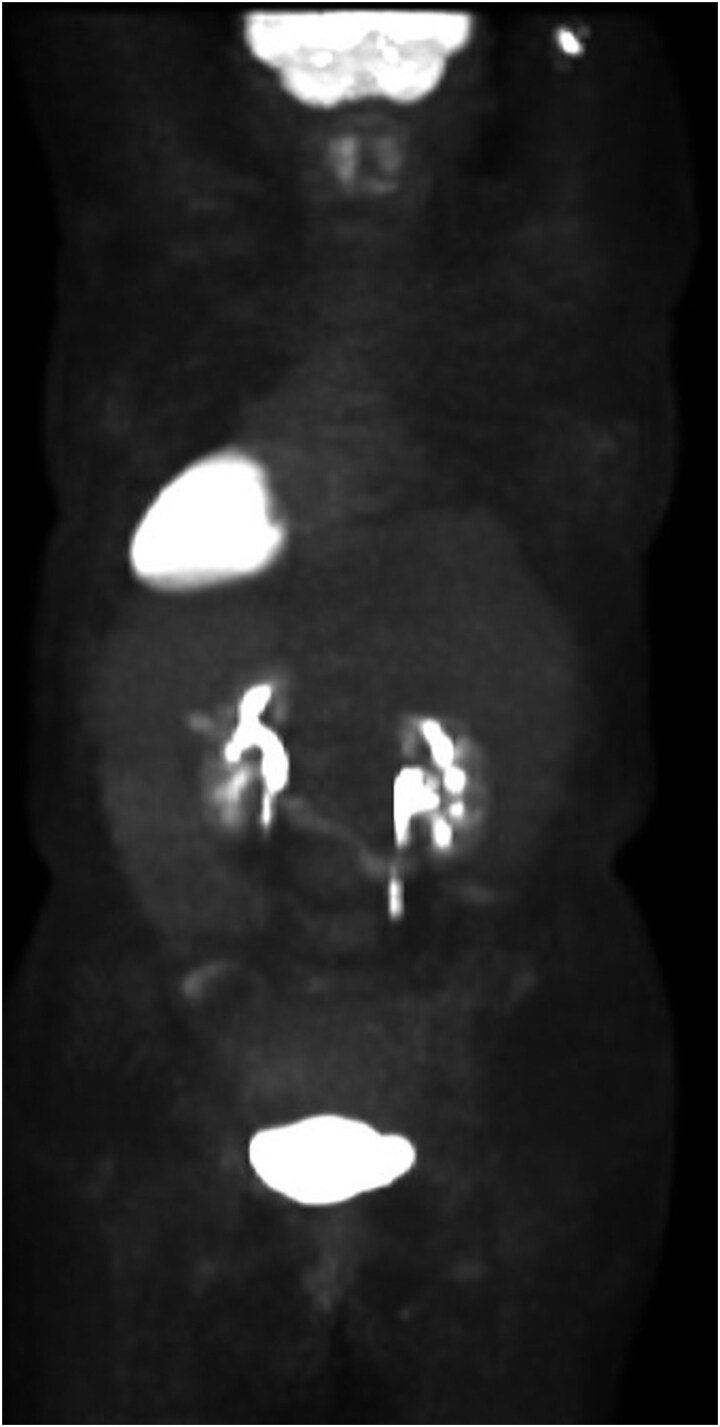
Patient’s preoperative PET scan, which demonstrates no significant hypermetabolic parenchymal lesions, lymph nodes, or masses.

**Figure 3:**
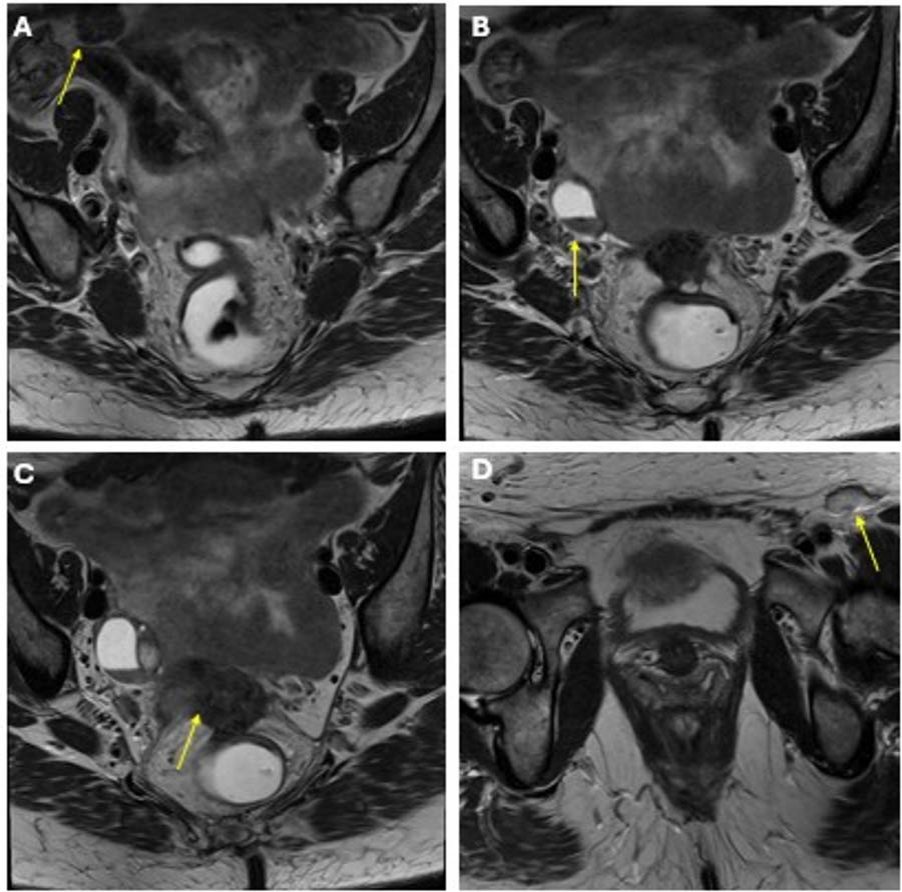
MRI obtained status post 20 cycles of adjuvant pembrolizumab with a stable-appearing mass abutting the right rectus abdominis (A), right adnexal masses (B), a mass along the anterior peritoneal reflection abutting the rectum (C), and a left inguinal lymph node (D).

She completed six cycles of adjuvant leucovorin/folinic acid, 5-fluorouracil, and oxaliplatin (FOLFOX) before developing pelvic and perineal discomfort. CT imaging demonstrated suspicious masses of the right abdominal wall (Fig. 1A), right ovary (Fig. 1B), anterior rectum (Fig. 1C), and left inguinal lymph node (Fig. 1D). A fine needle aspiration of the peritoneum and cervix confirmed metastatic colorectal adenocarcinoma. Given the progression despite being on chemotherapy, a multidisciplinary discussion was made to initiate pembrolizumab. The patient completed 1 year of therapy (20 cycles), which she tolerated well. Restaging with esophagogastroduodenoscopy (EGD), colonoscopy, and positron emission tomography (PET) demonstrated stable disease without progression (Fig. 2). MRI imaging redemonstrated essentially stable appearing right rectus abdominus muscle mass measuring 2.1 × 1.6 × 2.2 cm (Fig. 3A), two suspicious ovarian lesions measuring 2.9 × 1.7 × 3.2 and 2.0 × 1.2 × 1.9 cm (Fig. 3B), an additional mass abutting the anterior peritoneal reflection measuring 3.7 × 2.9 × 3.1 cm (Fig. 3C), and prominent 12 mm left inguinal lymph node (Fig. 3D).

**Figure 4:**
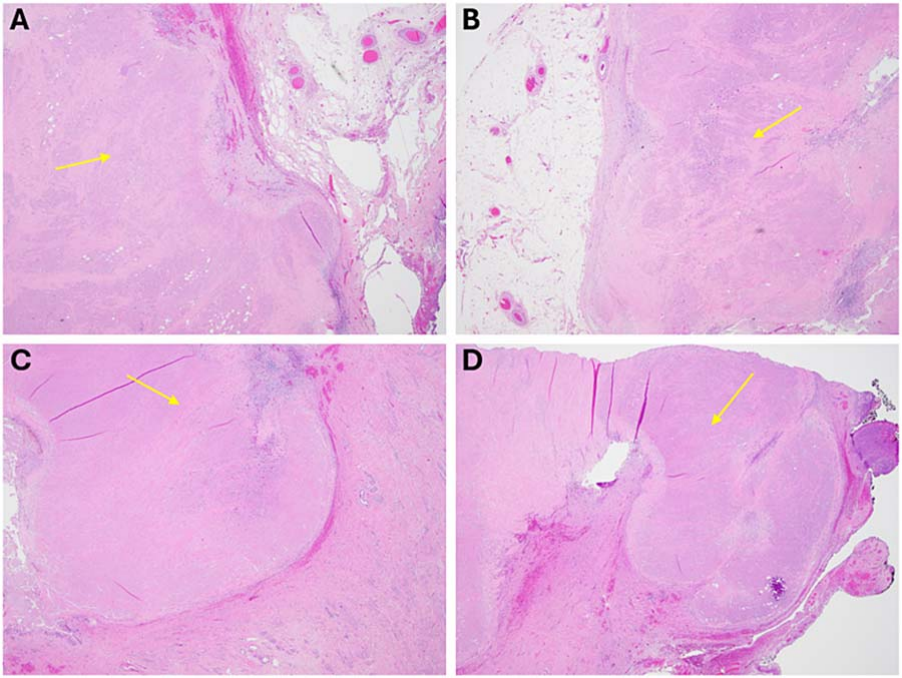
Final representative sections of the abdominal wall (A/B) and cervix (C/D) viewed at 2x power, demonstrating a pink, necrotic mass that is devitalized without viable tumour, with surrounding benign fibroadipose tissue and cervical stroma, respectively.

Following multidisciplinary review, she underwent end-colostomy reversal, total abdominal hysterectomy with bilateral salpingo-oophorectomy, omentectomy, and resection of the cul-de-sac mass. Final pathology revealed necrotic tissue with no evidence of viable malignancy of the abdominal wall (Fig. 4A and B) and cervix (Fig. 4C and D). She recovered well postoperatively and was advised to follow routine surveillance colonoscopy per the National Comprehensive Cancer Network (NCCN) guidelines.

## Discussion

Chemotherapy for locally advanced colon cancer was historically favoured due to improved overall survival when compared with surgery alone [2, 3]. However, these benefits were observed only in patients with proficient mismatch repair (pMMR) who received fluorouracil-based therapy [4]. dMMR or microsatellite instability–high (MSI-H) tumors—present in ~15%–20% of colorectal cancers [5]—are less responsive to conventional 5-fluorouracil or oxaliplatin-based regimens [6, 7]. For patients with dMMR/MSI-H disease, immunotherapy is now considered first-line treatment [8].

Pembrolizumab, a monoclonal antibody targeting PD-1, has been widely used in malignancies such as melanoma, non–small cell lung cancer, and head and neck squamous cell carcinoma since 2017. In KEYNOTE-164, pembrolizumab demonstrated sustained safety and efficacy in MSI-H patients who had previously received at least one line of chemotherapy [9]. Compared with chemotherapy, pembrolizumab showed superior progression-free survival (16.5 vs 8.2 months; *P* = 0.0002), higher complete or partial response rates (43.8% vs 33.1%), and fewer treatment-related complications (22% vs 66%) [1]. KEYNOTE-177, the 5-year follow-up study, demonstrated no significant difference in overall survival but confirmed durable tumor suppression and fewer adverse events, with 60% of patients crossing over from chemotherapy to immunotherapy [10].

Other immune checkpoint inhibitors—including nivolumab, toripalimab, and ipilimumab—have also shown promise in achieving complete or major pathological response in neoadjuvant settings [11, 12]. However, few reports describe pembrolizumab as an adjuvant therapy for recurrent colorectal cancer. Existing cases document radiologic complete responses in recurrences involving various organs, peritoneal surfaces, or lymph nodes [13–14]. To date, only one reported case has demonstrated a pathology-proven cPR in recurrent colorectal cancer following pembrolizumab therapy [15].

In the present case, the patient developed recurrence involving the uterus, peritoneal wall, and rectus muscle while on FOLFOX and was transitioned to pembrolizumab. Surgical resection following immunotherapy revealed a complete pathological response. This supports the potential utility of pembrolizumab as an adjuvant therapy in recurrent dMMR/MSI-H colorectal cancer, particularly in patients who have failed standard chemotherapy.

## Conclusion

Immunotherapy is now the standard of care for dMMR/MSI-H colorectal cancers [8]. Further studies are needed to evaluate pembrolizumab’s ability to induce cPR after progression on systemic chemotherapy. Although no consensus exists regarding optimal duration or endpoints of adjuvant pembrolizumab therapy, this case suggests that continuing treatment until definitive resection may help confirm a complete pathological response in recurrent disease.
